# Detection and Molecular Characteristics of *Toxoplasma gondii* DNA in Retail Raw Meat Products in Poland

**DOI:** 10.1089/fpd.2018.2537

**Published:** 2019-03-05

**Authors:** Jacek Sroka, Ewa Bilska-Zając, Angelina Wójcik-Fatla, Violetta Zając, Jacek Dutkiewicz, Jacek Karamon, Weronika Piotrowska, Tomasz Cencek

**Affiliations:** ^1^ Department of Parasitology and Invasive Diseases, National Veterinary Research Institute, Pulawy, Poland.; ^2^ Department of Health Biohazards and Parasitology, Institute of Rural Health, Lublin, Poland.

**Keywords:** Toxoplasma gondii, prevalence, raw meat products, PCR, genotyping, Poland

## Abstract

Raw and undercooked meat are regarded as important sources of *Toxoplasma gondii* infection of people in Europe; however, data concerning this issue in Poland are still insufficient. The aim of this study was to determine the prevalence of *T. gondii* DNA isolated from raw meat products retailed in Poland. The molecular characteristics of detected DNA were also performed. Samples of cured bacon, raw or smoked sausages, ham, and minced meat were examined for the presence of *T. gondii* DNA. Samples were digested by pepsin solution, followed by the DNA isolation. Nested and real-time polymerase chain reaction (PCR) was performed based on the amplification of 35-fold-repetitive *B1* fragment gene of *T. gondii*. For selected *B1*-positive samples, multiplex PCR was performed using *SAG1*, *SAG2* (*5′-SAG2* and *3′-SAG2*), *altSAG2*, *SAG3*, *GRA6*, *BTUB*, *C29-2*, and *L358* genetic markers. Amplicons were sequenced and analyzed with NCBI database. Among 3223 examined samples, 175 (5.4%) were PCR positive. The highest percentages of positive results were found for samples originating from south-east regions of Poland—Podkarpackie (17.9%), Małopolskie (12.6%), and Lubelskie (10.8%) (*p* < 0.001). The percentages of positive results for particular types of meat products—sausages, smoked meat products, ham, and minced meat—ranged from 4.5% to 5.8% and the differences between them were not significant (*p* > 0.05). Sequence analysis of selected *B1*-positive samples demonstrated mostly the alleles of clonal type III (49.0%), and less—type II (17.3%), and type I (10.2%) based on nine used genetic markers. The combinations of types I/II or II/III or I/III alleles at different loci were also found in 23.5% of cases. Detection of *T. gondii* DNA in raw meat products may indicate the potential health threat for consumers in Poland; however, for complete risk assessment of *T. gondii* infection, the additional studies, including detection of live parasite, are needed.

## Background

Toxoplasmosis, caused by an obligatory intracellular protozoan parasite *Toxoplasma gondii*, may pose a severe medical problem in a congenital form, as cerebral and ocular damage in newborns, and as an acquired infection in immunocompromised individuals (Dubey and Beattie, 1988). Toxoplasmosis has been also associated with behavioral changes and the development of psychiatric disorders (Flegr, 2007). *T. gondii* can infect humans and many species of warm blooded animals (Da Silva *et al.*, 2005). The prevalence of *T. gondii* infection in humans varies depending on age, geographical location, nutritional habits, and keeping of hygienic standards (Tenter *et al.*, 2000).

Toxoplasmosis has been demonstrated as a foodborne infection of global concern, posing the greatest disease risk among all parasitic infections (WHO, 2015; Limon *et al.*, 2017; Bouwknegt *et al.*, 2018). Fresh pork meat and meat products are regarded as one of the important risk factors of *T. gondii* infection, since viable *T. gondii* parasites have been isolated thereof (van der Giessen *et al.*, 2007; Kijlstra *et al.*, 2009; Limon *et al.*, 2017). By contrast, little is known about the presence of *T. gondii* in cured meat products.

*T. gondii* has a clonal populational structure (Ajzenberg, 2010) and most of the parasite isolates in Europe and North America are classified into three clonal lineages (type I–III) (Howe and Sibley, 1995; Ajzenberg *et al.*, 2002). Other clonal lineages were found in wildlife in North America (Khan *et al.*, 2011). In South America, *T. gondii* isolates are more genetically diverse, what can be a result of high biodiversity, geographical differences, and the important role of recombination in strain diversification (Lehmann *et al.*, 2006; Dubey *et al.*, 2008b; Pena *et al.*, 2008).

In Poland, the seropositivity of *T. gondii* in humans is estimated up to 60–70%, depending on a tested group (Nowakowska *et al.*, 2006b). The overall incidence of human toxoplasmosis in Poland is underestimated as only the congenital cases are recorded (17 cases in 2017; NIH Raport, 2017). This represents a small proportion of the total number of clinical (i.e., lymphadenopathy, chorioretinitis, and neurotoxoplasmosis) and asymptomatic cases. The identification of a possible correlation between the severity or type of disease and strain genotype might be important for determining the proper treatment and possible outcome of the disease in human *T. gondii* infection cases. However, there is no clear opinion so far, on the correlation between *T. gondii* strain genotype and character of symptoms in infected people (Fuentes *et al.*, 2001). So far, only few studies in Poland applied the genotyping of *T. gondii*. Nowakowska *et al.* (2006a) genotyped *T. gondii* isolated from infants with congenital toxoplasmosis and demonstrated the presence of type II. Dubey *et al.* (2008a) detected the nonclonal strains of *T. gondii* in chickens in Poland. In the study by Lass *et al.* (2012), genotyping of *T. gondii* oocysts found on fruits and vegetables showed types I and II at *SAG2* locus. Recently, our own studies showed high prevalence of type III in goats' milk (Sroka *et al.*, 2017).

Since pork is widely consumed in Europe, determining the frequency of rate of *T. gondii* infection in pigs and pork is very important for the prognosis of disease risk. The prevalence of *T. gondii* infection in pigs in Poland varies depending on type of housing and production system. Recent serological studies performed by the National Veterinary Institute in Pulawy (Poland), in 3600 pigs, showed positive results in 11.1–14.3% of examined animals (Sroka *et al.*, 2011; Sroka *et al.* unpublished data).

Aiming to continue our previous research concerning the prevalence of *T. gondii* in slaughtered animals in Poland, we chose, as a goal of this study, to determine the prevalence and genetic assortment of *T. gondii* strains in raw meat products from retail stores in Poland, in aspect of potential threat to human health.

## Materials and Methods

### Meat products samples

**FIG. 1.**
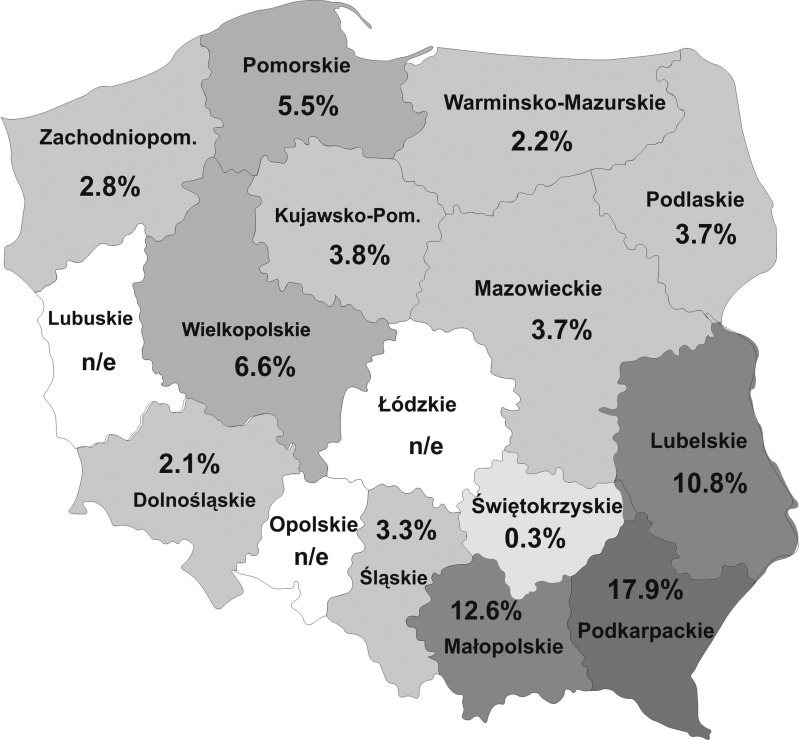
Results of *Toxoplasma gondii* DNA detection in raw meat products from selected regions of Poland.

The research was conducted as a part of the surveillance program realized by the National Veterinary Institute in Pulawy (Poland) (2014–2018). Samples were collected from the meat producers and retail meat stores offering raw meat products, randomly selected in each of 13 analyzed regions of Poland (Table 1; Fig. 1). The number of samples obtained in each of the regions depended on the availability of meat plants and retail stores offering raw meats products, as well as on the size of their assortment.

**Table 1.** Results of *Toxoplasma gondii* DNA Detection in Meat Products Retailed in Poland

*Type of meat products*	*Results for particular regions of Poland*													*Total*
	N *of positive/*N *of examined samples*													
	% of positive (CI)^*^													
	*Mp*	*Pk*	*Lb*	*Pl*	*W-m*	*Pm*	*Wk*	*Dl*	*Śl*	*Św*	*Zp*	*M*	*Kp*	
Smoked meat products	9/74	11/61	8/95	3/77	3/85	3/23	2/42	1/72	1/61	1/87	2/48	4/79	0/52	48/856
	12.2% (6.5–21.5)	18.0% (10.4–29.5)	8.4% (4.3–15.7)	3.9% (1.3–10.9)	3.4% (1.2–9.9)	13% (4.5–32.1)	4.8% (1.3–15.8)	1.4% (0.3–7.5)	1.6% (0.2–8.7)	1.1% (0.2–6.2)	4.2% (1.2–14.0)	5.1% (2–12.3)	0.0% (0.0–6.9)	5.7% (4.3–7.4)
Sausages	18/101	18/93	5/65	4/112	3/201	4/66	10/98	5/92	0/77	0/156	2/70	5/143	5/81	79/1355
	17.8% (11.6–26.4)	19.4% (12.6–28.5)	7.7% (3.3–16.8)	3.6% (1.4–8.8)	1.5% (0.5–4.3)	6.1% (2.4–14.6)	1.0% (5.6–11.8)	5.4% (5.2–11.6)	0.0% (0.0–4.8)	0.0% (0.0–2.4)	2.9% (0.8–9.8)	3.5% (1.5–7.9)	6.2% (2.7–13.7)	5.8% (4.7–7.2)
Ham	3/16	9/31	2/19	n/e	0/26	0/9	0/28	0/20	0/9	0/34	0/18	0/19	0/27	14/256
	18.8% (6.6–43.0)	29.0% (16.1–46.6)	10.5% (2.9–31.4)		0.0% (0.0–12.8)	0.0%/(0.0–29.9)	0.0% (0.0–12.1)	0.0% (0.0–16.1)	0.0% (0.0–29.9)	0.0% (0.0–10.2)	0.0% (0.0–17.6)	0.0% (0.0–16.8)	0.0% (0.0–12.5)	5.5% (3.3–9.0)
Minced meat	3/70	4/50	10/53	2/55	3/102	0/30	2/45	0/100	7/94	0/100	0/6	n/e	3/51	34/756
	4.3% (1.5–11.9)	8.0% (3.2–18.8)	18.9% (10.6–31.4)	3.6% (0.6–13.6)	2.9% (1.0–8.3)	0.0% (0.0–11.4)	4.4% (1.2–14.8)	0.0% (3.7–14.6)	7.4% (3.7–14.6)	0.0% (0.0–3.7)	0.0% (0.0–39.0)		5.9% (2.0–15.9)	4.5% (3.3–9.0)
Total	33/261	42/235	25/232	9/244	9/414	7/128	14/213	6/284	8/241	1/377	4/142	9/241	8/211	175/3223
	12.6%^*^ (9.1–17.2)	17.9%^*^ (13.5–23.3)	10.8%^*^ (7.4–15.4)	3.7% (2.0–6.9)	2.2% (1.2–4.1)	5.5% (2.7–10.9)	6.6% (4.0–10.7)	2.1% (1.0–4.5)	3.3% (1.7–6.4)	0.3% (0.0–1.5)	2.8% (1.1–7.0)	3.7% (2.0–7.0)	3.8% (2.0–7.3)	5.4% (4.7–6.3)

^*^ Significant differences in percentages of positive results in comparison with other regions of Poland (*p* < 0.05).

The regions of Poland: Mp, Małopolskie; Pk, Podkarpackie; Lb, Lubelskie; Pl, Podlaskie; W-m, Warmińsko-Mazurskie; Pm, Pomorskie; Wk, Wielkopolskie; Dl, Dolnośląskie; Śl, Śląskie; Św, Świętokrzyskie; Zp, Zachodniopomorskie; M, Mazowieckie; Kp, Kujawsko-pomorskie.

n/e, not examined; CI, 95% confidence interval.

In total, 3223 meat product samples were tested, including 2467 samples purchased (2015–2017) in retail meat stores and 756 samples collected by Veterinary Inspection in meat plants from 13 regions of Poland. The following kind of meat products were tested: raw sausages (i.e., Polish sausage, white sausage, Frankfurters, minced “Metka” sausage, kindikas, salami, “Cracow” dry sausage, thin dry-smoked pork sausage, juniper dry sausage, and hunter's sausage), raw or smoked bacon, smoked ribs, raw loin or tenderloin, raw (smoked) fermented ham, and minced meat.

### Digesting of meat product samples and DNA extraction

Meat product samples were digested by pepsin solution according to the method described by Dubey and Beattie (1988). Briefly, 50 g samples were cut and homogenized in 125 mL of 0.9% NaCl. Next, the homogenate of each sample was mixed with 250 mL of acid-pepsin solution (2.6 g pepsin, 7 mL HCl, and 0.9% NaCl filled up to 500 mL, pH 1.1–1.2), incubated in a shaker bath at 37°C for 90 min., poured out through the gauze, and centrifuged at 1200 × *g* for 10 min. The collected pellet was resuspended in 35 mL of phosphate-buffered saline (pH 7.4) and centrifuged (1200 × *g* × 10 min.). Supernatant was removed and pellet was resuspended in 5 mL of 0.9% NaCl, and stored at −20°C until further analysis. Genomic DNA was extracted from the pellet using a commercial kit (QIAmp DNA Mini Kit; Qiagen), according to the manufacturer's instructions.

### Nested and real-time polymerase chain reaction

Briefly, *T. gondii* DNA was detected by amplification of 35-fold-repetitive *B1* fragment gene in nested polymerase chain reaction (PCR) (according to the method by Grigg and Boothroyd, 2001) and, as an alternative technique, to confirm *B1* PCR-positive results, by real-time PCR (according to the method by Lin *et al.*, 2000). In real-time PCR, the commercial master mix QI Supermix (Bio-Rad, Hercules, CA) was used.

### Multilocus PCR

To investigate the clonal type of *B1* gene-positive samples, multilocus PCR was carried out using nine markers: *SAG1*, *3′SAG2*, *5′SAG2*, *SAG3*, *BTUB*, *GRA6*, *altSAG2*, *C29-2*, and *L358*, based on the method described by Su *et al.* (2010).

Nested and multilocus PCR were carried out in a C1000 Thermal Cycler (Bio-Rad). Real-time PCR amplification was performed in a thermal cycler CFX-96 (Bio-Rad). As positive controls, RH (type I), ME49 (type II), and C56 (type III) DNA isolates of *T. gondii* strains, and as a negative control nuclease-free water, were used.

DNA sequencing of amplicons was performed using ABI PRISM 310 Genetic Analyzer (Applied Biosystems, Inc., Foster City, CA), with the use of Abi Prism Big Dye Terminator v. 3.1. Cycle Sequencing Kits and Big Dye XTerminator Purification Kit (Applied Biosystems). Sequences were analyzed using Geneious v. 11.1.4. software (Geneious Co., Wellington, New Zealand) and compared with the sequences deposited in NCBI database using Blast.

### Statistical analysis

The results were analyzed with χ^2^ test, using STATISTICA v. 5.1 package (Statsoft, Tulsa, OK).

## Results

### PCR results

In total, among 3223 meat products samples examined, 175 (5.4%) were PCR positive, including 48 smoked meat products (27.4% of total positive), 79 sausages (45.1%), 14 hams (8.0%), and 34 minced meat samples (19.4%). All *B1* PCR-positive samples were positive in RT PCR; however, for part of samples (62 out of 175 samples), the borderline results (Ct values exceeded 40) were obtained. The highest percentages of positive results were obtained for samples originating from south-east regions of Poland—Podkarpackie (Pk) (17.9%), Małopolskie (Mp) (12.6%), and Lubelskie (Lb) (10.8%). The percentages of positive results for samples originating from other regions of Poland were significantly lower (0.3–6.6%) (*p* < 0.001) (Table 1). In individual groups of meat products, *T. gondii* DNA was detected in 5.7% of smoked meat products samples, 5.8% of sausage samples, 5.5% of ham samples, and 4.5% of minced meat samples; the differences between percentages were not significant. In the group of smoked meat products, the highest percentages of positive results were obtained for samples from Pk and Mp regions (18.0% and 12.2%), lower for samples from Pm and Lb regions (13% and 8.4%), and lowest for other regions (0.0–5.1%) (*p* < 0.05). In the group of sausages, significant differences were found between percentages of positive results obtained for samples from Pk and Mp regions (19.4% and 17.8%) in comparison with other regions (0.0–7.7%) (*p* < 0.05) The positive results for ham samples were obtained only for samples originating from three regions—Pk (29.0%), Mp (18.8%), and Lb (10.5%). Minced meat samples from Lb (18.9%) were significantly (*p* < 0.05) more likely to be PCR positive than samples from other regions (0.0–8.0%) (Table 1).

### Genotyping results

Among 175 DNA samples positive in PCR (*B1*), we were able to sequence 61 samples (in RT PCR, for these samples, Ct value did not exceed 39). The remaining samples did not have enough DNA amount to be processed for sequencing, or sequences were not of high quality for analysis.

Among DNA meat product samples, one sample was able to be genotyped with six markers (*SAG1*, *3′SAG2*, *SAG3*, *GRA6*, *altSAG2*, and *L358*), one sample with four markers (*5′SAG2*, *SAG3*, *GRA6*, and *altSAG2*), and five samples with three markers. Next, 19 samples were genotyped with two markers. The remaining sequenced samples (35) were positive only with one marker (7 samples with *SAG1*; 11 samples with *SAG3*; 9 samples with *GRA6*; 1 sample with *BTUB*, 1 sample with *3′SAG2*, 4 samples with *5′SAG2*, and 2 samples with *C358*). In total, by using nine markers, 98 amplicons were obtained (Table 2).

**Table 2.** Distribution of *Toxoplasma gondii* Genotypes in Sixty-One Meat Products Samples Included in the Study

*Pattern of possible amplifications*	*Total number of meat products DNA samples*	*SAG1*^a^	*3′SAG2*	*5′SAG2*	*SAG3*	*GRA6*	*BTUB*	*AltSAG2*	*C29-2*	*L358*
1	1	n/a	n/a	III	n/a	II	n/a	—	—	—
	1			II		III		n/a	n/a	n/a
2	1	II/III	III	n/a	III	n/a	n/a	—	—	—
3	1	II/III	III	III	n/a	n/a	n/a	—	—	—
4	1	I	n/a	n/a	III	n/a	n/a	—	—	—
	2	II/III			III					
5	1	I	n/a	III	n/a	n/a	n/a	—	—	—
	1	II/III		I/II^b^						
	1	II/III	n/a	II				n/a	n/a	n/a
6	1	n/a	n/a	III	III	n/a	n/a	—	—	—
7	1	n/a	n/a	n/a	III	I/II^b^	n/a	—	—	—
	1				I/III^b^	III		n/a	n/a	n/a
	1				II	III		n/a	n/a	n/a
	2				III	III		—	—	—
8	1	n/a	n/a	n/a	III	n/a	I/III^b^	—	—	—
9	6	II/III	n/a	n/a	n/a	n/a	n/a	-/n/a	-/n/a	-/n/a
	1	II/III						n/a	n/a	n/a
10	6	n/a	n/a	n/a	III	n/a	n/a	—	—	—
	2				I					
	3				II					
11	1	n/a	n/a	n/a	n/a	I/II^b^	n/a	—	—	—
	3					II		-/n/a	-/n/a	-/n/a
	5									
						III		-/n/a	-/n/a	-/n/a
12	2	n/a	n/a	I/II^b^	n/a	n/a	n/a	—	—	—
	2			III				-/n/a	-/n/a	-/n/a
13	1	n/a	n/a	n/a	n/a	n/a	I/III^b^	—	—	—
14	1	I	II	n/a	II	I	n/a	III	n/a	II
15	1	n/a	n/a	III	III	III	n/a	n/a	n/a	n/a
16	1	n/a	n/a	III	III	III	n/a	III	n/a	n/a
17	1	n/a	n/a	n/a	III	III	n/a	n/a	n/a	II
18	1	n/a	n/a	n/a	II	n/a	n/a	II	n/a	n/a
19	1	n/a	n/a	n/a	I	n/a	n/a	n/a	I	n/a
	1				II				III	
20	1	n/a	I/III^b^	n/a	n/a	n/a	n/a	n/a	n/a	n/a
21	2	n/a	n/a	n/a	n/a	n/a	n/a	n/a	n/a	III
22	1	I	n/a	I/II^b^	I	n/a	n/a	n/a	n/a	n/a
23	1	n/a	n/a	n/a	III	n/a	n/a	n/a	n/a	III

GenBank accession numbers for selected sequences: MH429059- MH429071, MH536007- MH536012, and MH606148–MH606184.

^a^ At SAG1 locus, types II and III are indistinguishable.

^b^ Distinction between these types was not possible, probably a mix of *T. gondii* strains occurred.

—, not examined; n/a, product not amplified; -/n/a, part of samples not examined, for next part of samples − product not amplified.

In total, type I, type II, and type III *T. gondii* lineages were determined for 10 (10.2%), 17 (17.3%), and 48 (49.0%) amplicons, respectively. Alleles types I/II, II/III, and I/III had 6 (6.1%), 13 (13.3%), and 4 (4.1%) amplicons, respectively (Table 3). The new single-nucleotide polymorphisms (SNPs) were found in three amplicons.

**Table 3.** The Summary of *Toxoplasma gondii* Genotypes Determined at Individual Markers Used in Study

*Genotype markers*	N *of positive DNA samples*	*Type I*	*Type II*	*Type III*	*Type I/II*^a^	*Type II/III*^a^	*Type I/III*^a^
*SAG1*	17	4	0	0	0	13	0
*3′SAG2*	4	0	1	2	0	0	1
*5′SAG2*	14	0	2	8	4	0	0
*SAG3*	31	4	7	19	0	0	1
*GRA6*	20	1	4	13	2	0	0
*BTUB*	2	0	0	0	0	0	2
*altSAG2*	3	0	1	2	0	0	0
*C29-2*	2	1	0	1	0	0	0
*L-358*	5	0	2	3	0	0	0
Total	98	10^b^ (10.2%)	17^b^ (17.3%)	48^b^ (49.0%)	6^b^ (6.1%)	13^b^ (13.3%)	4^b^ (4.1)

^a^ Distinction between these types was not possible, probably a mix of *T. gondii* strains occurred.

^b^ *N* of *T. gondii* DNA samples (percentage).

In relation to the genotyping results of particular amplicons and the regions of study, the high numbers of *T. gondii* type III and mix of types II/III were determined for samples from Mazowieckie and Wielkopolskie (Wk, Type III), and Mp (types II/III) regions (*p* < 0.05), whereas, type I was mostly detected in samples from Mp and Zp regions (Table 4).

**Table 4.** The Summary of Genotyping in Relation to the Regions of Study (Poland)

*Regions of study*	N *of positive DNA samples*	*Type I*	*Type II*	*Type III*	*Type I/II*^a^	*Type II/III*^a^	*Type I/III*^a^
Kujawsko-Pomorskie	9	0	4	5	0	0	0
Małopolskie	40	5	1	18	3	11^b^	2
Mazowieckie	10	0	2	7^b^	0	0	1
Podkarpackie	13	0	4	6	2	1	0
Wielkopolskie	14	1	3	7^b^	1	1	1
Zachodniopomorskie	12	4	3	5	0	0	0
Total	98	10 (10.2%)	17 (17.3%)	48 (49.0%)	6 (6.1%)	13 (13.3%)	4 (4.1%)

^a^ Distinction between these types was not possible, probably a mix of *T. gondii* strains occurred.

^b^ Significant differences in the number of genotypes determined for particular amplicons, in comparison with other regions of Poland (*p* < 0.05).

Taking into account only the results with homogenous genotype obtained for particular meat product samples, finally, type III was determined for 21, type II for 7 samples, and type I for 3 samples. The highest number of meat product samples with *T. gondii* type III genotypes was detected in Mp region; however, this difference did not attain a level of statistical significance (Table 5).

**Table 5.** The Number of Meat Product Samples with the Homologous Genotypes Obtained at Used Markers, in Relation to the Regions of Study

*Regions of Poland*	*Type I*	*Type II*	*Type III*	*Total*
Kujawsko–Pomorskie	0	2	1	3
Małopolskie	2	0	7	9
Mazowieckie	0	0	3	3
Podkarpackie	0	3	4	7
Wielkopolskie	0	2	3	5
Zachodniopomorskie	1	0	3	4
Total	3 (9.7%)	7 (22.6%)	21 (67.7%)	31

The comparison of genotyping results in selected 61 meat products, in relation to the type of meat products, showed the high number of type II and type I in minced meat samples (beef), whereas type III was prevalent in sausages and pork minced meat samples (Table 6).

**Table 6.** The Summary of Genotyping in Sixty-One Meat Products Samples, in Relation to Their Types

*Types of meat products*	*N of positive DNA samples*	*Type I*	*Type II*	*Type III*	*Type I/II*^a^	*Type II/III*^a^	*Type I/III*^a^
Sausages	37	5	4	20	3	3	2
Smoked meat products	24	0	2	12	0	9	1
Hams	7	0	2	2	2	0	1
Minced meat (total), including	30	5	9^b^	14	1	1	0
Pork	8	0	0	7^b^	0	1	0
Beef	22	5	9	7	1	0	0
Total	98	10 (10.2%)	17 (17.3%)	48 (49.0%)	6 (6.1%)	13 (13.3%)	4 (4.1%)

^a^ Distinction between these types was not possible, probably a mix of *T. gondii* strains occurred.

^b^ Significant differences in the number of genotypes determined for particular amplicons, in comparison with other types of meat products or in a particular group of meat products (*p* < 0.05).

## Discussion

The foodborne route of human *T. gondii* infection is mainly linked with consumption of raw or undercooked meat and meat products (Kapperud *et al.*, 1996; Cook *et al.*, 2000; WHO, 2015; Jones *et al.*, 2009). Serological examination in pigs, in general, is useful for indirect diagnosis of *T. gondii* infection. Seropositivity in pigs can indicate parasite presence in their tissues. Some authors report that the success of live *T. gondii* isolation increases with antibody titer in the pig (Dubey *et al.*, 1995), whereas the others have not found such correlation. In a recent study, Kuruca *et al.* (2017) detected *T. gondii* DNA in diaphragm tissues of eight pigs, of which three were seronegative. In the study by Dubey *et al.* (2012), live *T. gondii* strains were isolated from 17 pigs, including one from a seronegative animal. Thus, meat from seronegative pigs may also represent a source of potential infection to consumers.

Recent trends in consumer habits, with a shift toward the consumption of free-range and organic pork, where animals have a higher risk of exposure to *T. gondii* from the environment, may result in a higher risk of consumer exposure to *T. gondii* (van der Giessen *et al.*, 2007; Kijlstra *et al.*, 2009). Thus, the genotyping of *T. gondii* strains isolated from various sources of infection may provide new insights to evaluate the possible interaction between parasite types and severity of the disease in humans (Da Silva *et al.*, 2005).

In Poland, there are still regions with small, family farms with traditional rearing of pigs, which have direct contact with potential sources of *T. gondii*. Having outdoor access, the presence of cats in farm, and feed stored with the possibility for contamination with cat feces have been previously reported as risk factors for *T. gondii* infection (Assadi-Rad *et al.*, 1995; Weigel *et al.*, 1995; Kijlstra *et al.*, 2004; Klun *et al.*, 2006; Guo *et al.*, 2016).

In this context, noteworthy are regional differences between the frequency rates of *T. gondii* in meat samples, stated by us in this study. The highest prevalence of *T. gondii* DNA was found in two regions (Małopolskie and Podkarpackie) situated in the southeastern part of Poland. These are mountainous regions with numerous small farms, where pigs are often free-range reared and might have direct contact with cats, wild animals, and other potential sources of *T. gondii* infection.

Basic traditional methods of the production of Polish hams and sausages are based on drying (hanging until mature), followed by smoking. Especially, Śląskie, Podlaskie and Wielkopolskie are the regions in Poland where raw sausage, ham, and smoked meat products are produced according to traditional, Polish recipes. There are preserved with salt only, then smoked, and dried.

In this study, a total of 5.4% tested raw meat products were detected, *T. gondii* positive, mainly sausage (45.1% of total positive), less smoked meat products (27.4%), and least ham (8.0%). The other reports from European countries may confirm our results; Aspinall *et al.* (2002) in a study conducted in United Kingdom showed 38% of meat and meat product samples contaminated with *T. gondii*. Vergara *et al.* (2018) detected *T. gondii* DNA in carcasses of 14 out of 103 pigs (13.6%) at an abattoir in Italy. In a study performed in Turkey (Ergin *et al.*, 2009), *T. gondii* DNA was detected in 19% of fermented sausages. The possibility of live *T. gondii* persistence in meat products was reported by Warnekulasuriya *et al.* (1998), who detected viable *T. gondii* organisms in one of 67 cured meat samples from United Kingdom, including dried and semidried sausages and hams. In the studies performed in the New World, the prevalence of *T. gondii* in meat samples ranged in fairly wide limits from 0.5% to 43% (Dias *et al.*, 2005; Dubey *et al.*, 2005; Galvan-Ramırez *et al.*, 2010; Franco-Hernandez *et al.*, 2016).

There is little knowledge concerning the effects of several processing conditions during preparation of meat products on inactivation of parasite, such as salt, nitrites, nitrates, and organic acid concentration, time, and temperature processing. In opinions of some researchers, tissue cysts of *T. gondii* are killed during commercial curing procedures with salt (Dubey, 1997; Hill *et al.*, 2004, 2006; Genchi *et al.*, 2017). In contrast, other studies have indicated the potential failure of curing to inactivate *T. gondii* (Warnekulasuriya *et al.*, 1998; Herrero *et al.*, 2017).

In this study, the clonal *T. gondii* type III was the most prevalent, while types I and II were less common. This finding is concordant with the report by Zia-Ali *et al.* (2007) from Iran, who found *T. gondii* type III (*SAG2*) in the majority of tested tissues samples of chicken and sheep. Similarly, Lehmann *et al.* (2003) found 25 *T. gondii* isolates from market-age pigs in the United States of America, 20 belonging to type III and 5 to type II. In the study performed in Italy, Vergara *et al.* (2018) identified in pigs types III (3.9%), I (3.9%), and II (5.8%). However, in other European studies, *T. gondii* type II isolates appeared to be most common (Richomme *et al.*, 2009; Aubert *et al.*, 2010). By contrast, in some genotypic studies performed in United Kingdom and South America, prevalence of type I was stated among *T. gondii* strains isolated from meat samples (Aspinall *et al.*, 2001; Da Silva *et al.*, 2005).

Since molecular methods for detecting *T. gondii* DNA were used, we were unable to distinguish viable from nonviable parasites. Because condiments (potential PCR inhibitors) are present in cured meat products, the efficiency of the amplification of *T. gondii* DNA from cured meat products may be problematic. Amplification failure with some markers might depend on the quality of tissue/DNA samples (Franco-Hernandez *et al.*, 2016). To gain good efficiency of marker amplification, a good quality DNA is needed; however, this is not always possible in epidemiological studies.

In this study, some of *B1* PCR-positive samples failed to be completely genotyped and only partial data were obtained due to a low DNA concentration. Considering this limitation, a cautious presumption could be made that genotype III seems to be the most prevalent in the analyzed area followed by genotype II and I, while alleles types I/II, II/III, and I/III had lower prevalence. For three DNA samples, new SNPs were also found. These results seem to be different from those obtained in other European countries, in which *T. gondii* genotype II was the most widespread. The prevalence of *T. gondii* genotype II ranged in Europe from 50% in Spain (Calero-Bernal *et al.*, 2015) to 100% in France (Richomme *et al.*, 2009; Aubert *et al.*, 2010). However, in some regions of Europe (i.e., Italy, Spain, Switzerland, Slovakia, and Portugal), the genetic variability can be higher than previously stated and type III, type I, and mixed or atypical strains of *T. gondii* may be more frequent (Fuentes *et al.*, 2001; de Sousa *et al.*, 2006; Berger-Schoch *et al.*, 2011; Mancianti *et al.*, 2013; Turčeková *et al.*, 2013; Verin *et al.*, 2013; Bacci *et al.*, 2015; Formenti *et al.*, 2016; Battisti *et al.*, 2018). In Poland, the results of our own, previous research, where types III and I, as well as mixed or atypical were detected (i.e., in goat milk, ticks from vegetative stage, and wildlife), may confirm this statement (Sroka *et al.*, 2016, 2017; Cisak *et al.*, 2017; Zając *et al.*, 2017). According to some authors' opinions (Xiao and Yolken, 2015), *T. gondii* type I and atypical strains can cause more severe disease symptoms, especially in immunocompromised persons.

## Conclusion

The results of this study showed that raw meat products seem to be potentially important source of *T. gondii* infection for humans in Poland, which may be important for persons with immunodeficiency and pregnant women, who should avoid eating raw meat product.

However, for a complete risk assessment, the additional studies, including detection of live parasites, are needed.
